# Internet-based cognitive behavioural therapy (iCBT) for posttraumatic stress disorder versus waitlist control: study protocol for a randomised controlled trial

**DOI:** 10.1186/s13063-015-1059-5

**Published:** 2015-12-01

**Authors:** Adrian R. Allen, Jill M. Newby, Jessica Smith, Gavin Andrews

**Affiliations:** Clinical Research Unit for Anxiety and Depression, School of Psychiatry, University of New South Wales at St Vincent’s Hospital, Level 4, O’Brien Centre, St. Vincent’s Hospital, 394-404 Victoria Street, Darlinghurst, NSW Australia

**Keywords:** Internet cognitive behavioural therapy, Posttraumatic stress disorder, Randomised controlled trial

## Abstract

**Background:**

This randomised controlled trial (RCT) with two parallel arms will evaluate the efficacy of an internet-delivered six-lesson 10-week cognitive behavioural therapy (iCBT) intervention for posttraumatic stress disorder (PTSD). It will also investigate the association between changes in PTSD symptoms, intolerance of uncertainty (IU) and emotion regulation.

**Methods/Design:**

Patients with PTSD will be recruited via the research arm of a not-for-profit clinical and research unit in Australia and randomised to a treatment group or waitlist control group. The minimum sample size for each group (alpha 0.05, power 0.80 for a *g* of 0.47) was identified as 72, but 10 % more will be recruited to hedge against expected attrition. PTSD diagnosis will be determined using the PTSD module from the Mini International Neuropsychiatric Interview version 5.0.0. The PTSD Checklist – Civilian version (PCL-C) will be used to measure PTSD symptoms (the primary outcome measure), with the Intolerance of Uncertainty Scale 12-item version (IUS-12) and the Emotion Regulation Questionnaire (ERQ) used to measure intolerance of uncertainty and emotion regulation, respectively. The PCL-C will be administered to the treatment group before each lesson of the PTSD program and at 3-month follow-up. The IUS-12 and ERQ will be administered before lessons 1 and 4, at post-treatment and at 3-month follow-up. The waitlist control group will complete these measures at week 1, week 5 and week 11 of the waitlist period. PTSD program efficacy will be determined using intent-to-treat mixed models. Maintenance of gains will be assessed at 3-month follow-up. Mediation analyses using PROCESS will be used to examine the association between change in PTSD symptoms over treatment and change in each of IU and emotion regulation ability in separate analyses.

**Discussion:**

The current RCT seeks to replicate previous efficacy findings of iCBT for PTSD in a formally assessed PTSD sample from the general population. Findings may point to future lines of enquiry for the role of IU and emotion regulation in the mechanism of PTSD symptom change during CBT.

**Trial registration:**

Australian New Zealand Clinical Trials Registry: ACTRN12614001213639, registered 18 November 2014. This trial protocol is written in compliance with the Standard Protocol Items: Recommendations for Interventional Trials (SPIRIT) guidelines.

## Background

Posttraumatic stress disorder (PTSD) can follow experiencing a traumatic event in which the individual experiences or witnesses severe threat to life or physical safety. It is characterised by recurrent intrusive thoughts or memories of the traumatic event, avoidance of trauma reminders, elevated physiological arousal and negative changes in thoughts and mood [1]. The lifetime prevalence of PTSD is approximately 7 % in the general population [2–4]. It has a chronic course when untreated – up to one third of those with PTSD remain symptomatic for 30 years [5]. The impact of the disorder on the individual is significant. Compared to those without the disorder, PTSD is independently associated with: greater prevalence of physical health conditions, such as respiratory disease, cardiovascular disease, cancer and gastrointestinal illnesses [6], but see [7]; increased comorbidity of mental health conditions, such as anxiety disorders and depression [8]; and increased risk of suicidal behaviour [9]. Taken together, these data indicate a significant individual impact from the disorder, as well as societal cost in terms of burden of disease.

Cognitive behavioural therapy (CBT) is the psychological treatment of choice for PTSD and is recommended by best-practice treatment guidelines e.g. [10, 11]. CBT typically involves confrontation with, and processing of, the trauma memory in a safe, gradual manner; identification and restructuring of problematic beliefs; and dearousal skills. There is strong research evidence for use of these CBT techniques to treat PTSD in terms of magnitude of symptom reduction from pre-treatment levels, and diagnostic recovery [12–15]. Despite this evidence, those with PTSD are an underserviced population. The majority of those with PTSD do not seek treatment and those seeking treatment do so with significant delay [16]. Associated treatment barriers include stigma, cost, geography and insufficient treatment availability [17]. Indeed, McLean and Foa [17] have noted that development of effective and efficient delivery systems for evidence-based PTSD treatments remains a key challenge in the trauma field.

Attempting to counter these difficulties, recent efforts have examined internet-based delivery of empirically supported treatment for PTSD symptomatology. Clinical research has shown that internet-based delivery of cognitive behavioural therapy (iCBT) has utility for treating PTSD symptoms to an extent approximately equivalent to face-to-face treatment [18–21]. These treatments are typically divided into five to ten lesson modules, delivered over several weeks, with some form of therapist support (e.g. email or phone).

Though iCBT appears promising in treating PTSD, variation in experimental design and course structure means further clinical trials are warranted. Moreover, the role of emotion process variables in PTSD response to iCBT has not been examined. To date, published studies have either not included a control group e.g. [21], not formally diagnosed PTSD e.g. [19], been based on a self-help mode of treatment e.g. [22] or required significant initial therapist input to provide psychoeducation and explain treatment components to the patient e.g. [18]. Only two published randomised controlled trials (RCT) to date have examined the efficacy of iCBT for PTSD in a formally diagnosed PTSD sample from the general population [20, 23]. However, those studies did not examine mediators of symptom change. Therefore, in order to extend and validate iCBT for PTSD, the proposed study will conduct an RCT to examine the clinical efficacy of this approach and mediators of PTSD symptom change.

Emerging evidence suggests intolerance of uncertainty (IU) as a transdiagnostic construct central to the expression of psychopathology [24–26]. IU is the tendency to respond to uncertainty with a negative emotional and cognitive set and corresponding avoidance and risk mitigation behaviour [27]. Recent research has indicated reduction in IU is associated with anxiety and depressive symptom reduction following psychological treatment [28]. With respect to PTSD, IU has been positively associated with severity of arousal, avoidance and numbing symptoms [29]. However, the association between PTSD treatment and variation in IU has not been investigated, nor has the relationship between change in IU and PTSD symptoms over treatment. Therefore, the current study will examine these relationships in PTSD.

Finally, recent work has investigated the role of emotion regulation in PTSD treatment outcome. Emotion regulation is the ability to actively influence the timing, experience and expression of emotion. Emotion regulation skills training has been shown to reduce dropout and enhance outcome from subsequent exposure-based PTSD treatment [30, 31]. Moreover, exposure-based treatment for PTSD has been associated with enhancement in emotion regulation [32]. However, it remains unclear whether emotion regulation before treatment predicts an individual’s response to treatment. While Wisco et al. [33] found better pre-treatment emotion regulation skills were not predictive of enhanced PTSD treatment outcome, that treatment omitted in vivo exposure and cognitive restructuring, treatment components to which emotion regulation may be conceptually related. The association between emotion regulation and treatment outcome in an internet-delivered intervention for PTSD comprising written exposure, in vivo exposure and cognitive restructuring has not been investigated. As such, this study seeks to examine the role of emotion regulation in PTSD symptom response to this treatment approach.

### Objectives

The current Standard Protocol Items: Recommendations for Interventional Trials (SPIRIT)-compliant [34] protocol outlines the methodology of a CONSORT-compliant [35] RCT, the primary objective of which is to establish the efficacy of the iCBT program for PTSD. Secondary objectives are to examine change in IU and emotion regulation ability following the iCBT program and the association between change in each of these constructs and PTSD symptom change following the PTSD program.

The proposed iCBT program aims to reduce psychological distress and PTSD symptoms in those with PTSD. It comprises psychoeducation about the trauma reaction and how it is maintained after the event by particular patterns of thinking, feeling and behaving. Dearousal skills are taught and practiced. Participants are taught ways to identify and change unhelpful thoughts about the trauma. They will be habituated to their challenging trauma memories using a series of brief structured writing assignments and taught graded exposure methods to re-enter feared situations. Finally, relapse prevention will cover continued skills practice and instruction on independent management of potential future setbacks. These elements have been incorporated into standard CBT-based treatment for PTSD both in internet and face-to-face formats [12, 18–21, 36–38], but have been adapted for the proposed internet treatment program.

Hypotheses are as follows: (1) patients receiving iCBT for PTSD will show greater mean symptom reduction on a standardised PTSD measure than a waitlist control group during treatment and at the end of treatment (the primary endpoint), with preserved symptom reduction at 3-month follow-up; (2) IU will be less at post-treatment than pre-treatment; (3) reduction in IU over treatment will be associated with reduction in PTSD severity; (4) emotion regulation will be greater at post-treatment than pre-treatment; and (5) increased emotion regulation after treatment will be associated with reduced PTSD severity after treatment.

## Methods/Design

### Study setting

The Clinical Research Unit for Anxiety and Depression (CRUfAD) is a non-profit joint initiative of St. Vincent’s Hospital and the University of New South Wales, School of Psychiatry in Sydney, Australia. CRUfAD specialises in the development, evaluation, and dissemination of evidence-based CBT programs via the internet. This RCT will be conducted within the Virtual Clinic, CRUfAD’s clinical research arm (www.virtualclinic.org.au). The mode of internet recruitment and delivery enables potential participants from all Australian states to apply for enrolment in the current trial.

### Participants and recruitment

**Fig. 1:**
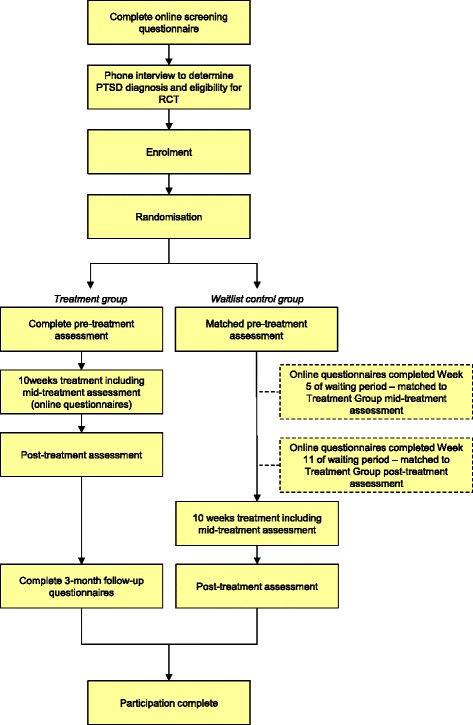
Participant flow chart

Participants will be recruited through flyers, paid print and internet advertising. Applicants will first complete online screening questionnaires about symptoms and demographic details (see Fig. 1 for a participant flow chart). Inclusion criteria are as follows: meet diagnostic criteria for PTSD; computer, internet and printer access; Australian resident; fluent in written and spoken English; willing to provide name, phone number and address, and to provide the name and address of a local general practitioner. Exclusion criteria are as follows: trauma occurrence within the past 4 weeks; non-resident of Australia; less than 18 years of age; currently receiving treatment for trauma/PTSD; frequent suicidal ideation (indicated by a score of 3 to item 9 of the Patient Health Questionnaire 9-item version (PHQ9)); regularly using illicit drugs or regularly consuming more than three standard drinks per day; psychotic disorder or taking atypical antipsychotics or benzodiazepines; if taking medication for anxiety or depression, has been taking the same dose for less than 1 month or intending to change the dose during the course of the program; currently highly dissociative (indicated by score ≥40 on the Dissociative Experiences Scale as per Spence et al. [20]); current or pending medicolegal proceedings associated with the reported trauma; applying for, or receiving, Workers Compensation associated with their trauma or PTSD. Excluded applicants will receive information on alternative services and will be encouraged to discuss their symptoms with their physician. Applicants who pass the screening phase will be telephoned for a diagnostic interview using the PTSD module from the Mini International Neuropsychiatric Interview version 5.0.0 (MINI) [39] to determine whether they meet diagnostic criteria for PTSD. All interviews will be conducted by a registered Clinical Psychologist (AA) or Clinical Trials Manager (JS) under the guidance of the Clinical Psychologist and trained in administration of the diagnostic interview. Applicants who satisfy all inclusion criteria will be informed of the study design and will complete an electronic informed consent prior to enrolling in the trial. Information from the diagnostic interview will be used for research purposes only for those participants who provide informed consent. All participants will be informed in writing that they may withdraw from the study (i.e. choose to cease program enrolment or choose for their data to be excluded from the RCT) at any time without jeopardising their relationship with St. Vincent’s Hospital or the University of New South Wales. Further, those participants randomised to the waitlist control group will be informed that should they commence a trauma-focused intervention outside of the trial, their data will be omitted from the RCT.

### Trial design and blinding

The trial is a randomised controlled superiority trial with two parallel arms using a 1:1 allocation ratio. Participants will not be blind to their group allocation. Follow-up interviews will be conducted by clinicians independent of the study and therefore blind to group allocation.

### Randomisation

Accepted participants will be randomised based on an allocation sequence generated by an independent person not involved in the study via a true randomisation process (www.random.org). Numbers corresponding to treatment group (1) or the waitlist control group (2) will be placed in sealed opaque envelopes bearing the sequential order number to ensure participant allocation based on the pre-determined randomisation sequence. A member of the research team will open the envelope after the diagnostic interview.

### Primary outcome measures

Administration time points for all instruments are contained in Table 1.

**Table 1:** Administration time points for questionnaires used in the RCT

	Administration time point				
Measure	Pre-treatment	Before each lesson	Mid -treatment^d^	Post-treatment^e^	3-month follow-up^f^
LEC^a^	♦				
DES	♦				
MINI	♦				♦
PCL-C^b^	♦	♦	♦	♦	♦
BDI-II^c^	♦		♦	♦	♦
PHQ-9	♦		♦	♦	♦
K10	♦	♦	♦	♦	♦
GAD-7	♦		♦	♦	♦
CEQ	♦			♦	
IUS-12	♦		♦	♦	♦
ERQ	♦		♦	♦	♦
SUDOR	♦		♦	♦	♦
SAPAS	♦				

*LEC* Life Events Checklist, *DES* Dissociative Experiences Scale, *MINI* Mini International Psychiatric Interview, *PCL-C* PTSD Checklist **–** Civilian version, *BDI-II* Beck Depression Inventory – Second Edition, *PHQ-9* Patient Health Questionnaire 9-item version, *K10* Kessler Psychological Distress Scale 10-item version, *GAD-7* Generalised Anxiety Disorder 7-Item Scale, *CEQ* - Credibility and Expectancy/Satisfaction Questionnaire, *IUS-12* Intolerance of Uncertainty Scale 12-item version, *ERQ* Emotion Regulation Questionnaire, *SUDOR* Service Utilisation and Days Out of Role Questionnaire, *SAPAS* Standardised Assessment of Personality - Abbreviated Scale

^a^Refers to both LEC-IV and part 2 of LEC-5

^b^Includes items 10, 11, and 16 from PCL-5

^c^Item 9 only

^d^Waitlist control group completes these measures at week 5 of the waitlist period

^e^Waitlist control group completes these measures at week 11 of the waitlist period

^f^Treatment group only

**Posttraumatic stress disorder Checklist – Civilian version** (PCL-C) [40]. The PCL-C is a 17-item self-report questionnaire providing a continuous measure of PTSD symptomatology, conforming to Diagnostic and Statistical Manual of Mental Disorders, Fourth Edition (DSM-IV) diagnostic criteria. Each item reflects a PTSD symptom and is rated on a 5-point scale for distress (1 = “Not at all”; 5 = “Extremely”). Items 10, 11 and 16 from the PCL-5 [41] will also be administered, which assess new diagnostic criteria for PTSD in the fifth edition of the DSM [DSM-5 1]. This approach was adopted in favour of sole use of the PCL-5, which had not been validated at the time of trial design. This will enable examination of change in the new PTSD symptoms in DSM-5.

**PTSD diagnostic status**. Change in PTSD diagnostic status will be indexed using the MINI PTSD module. The MINI has sound reliability and validity psychometrics [39].

### Secondary outcome measures

**Patient Health Questionnaire 9-item scale** (PHQ-9) [42]. The PHQ-9 is a self-report questionnaire corresponding to DSM-IV diagnostic criteria for major depressive disorder. Each item is rated in frequency on a 4-point scale (0 = “Not at all”; 3 = “Nearly every day”). Total scores range from 0 to 27 with higher scores reflecting greater symptom severity. The PHQ9 demonstrates sound psychometric properties and has been used extensively to measure treatment outcomes during internet CBT interventions targeting depression and anxiety [43, 44].

**Kessler-10 Psychological Distress Scale** (K10) [45]. The K10 consists of 10 items ranked on a 5-point scale designed to measure non-specific psychological distress. For the current study, the time frame was modified to assess psychological distress in the past 2 weeks rather than in the past 30 days. The K10 possesses strong psychometric properties [45, 46].

**Generalised Anxiety Disorder 7-Item Scale** (GAD-7) [47]. The GAD-7 is composed of seven items examining GAD symptom severity and is based on DSM-IV criteria. Items are measured on a 4-point scale for frequency of interference from symptoms over the past 2 weeks (0 = “Not at all”; 3 = “Nearly every day”). Internal consistency and test-retest reliability are high [47, 48].

**Intolerance of Uncertainty Scale – Short Form** (IUS-12) [49]. The IUS-12 is a psychometrically reliable, valid measure of intolerance of uncertainty [49]. It contains 12 items, each measured on a 5-point scale from 1 (“Not at all characteristic of me”) to 5 (“Entirely characteristic of me”).

**Emotion Regulation Questionnaire** (ERQ) [50]. The ERQ is a 10-item self-report measure of cognitive reappraisal and emotional suppression as means of regulating emotion. Items are scored on a 7-point scale to represent their use by the individual from 1 (“Strongly disagree”) to 7 (“Strongly agree”). This measure has sound reliability (including test-retest reliability) [51].

**Service Use and Days Out of Role Questionnaire - modified** (SUDOR) [52]. The SUDOR contains three items that measure the impact of symptoms on daily functioning including doctor (or other health professional) visits and days out of role or reduced functioning due to symptom impact. Items are presented in the form of open questions. Four additional items were included enquiring as to medication use and additional current treatment or activities undertaken to manage symptoms.

**Credibility and Expectancy/Satisfaction Questionnaire** (CEQ) adapted from [53]. At baseline, participants will complete two treatment expectancy questions: (1) “At this point, how logical does the program offered to you seem?” (0 = “Not at all logical”; 9 = “Very logical”) and (2) “At this point, how successfully do you think this treatment will be in reducing your PTSD symptoms?” (0 = “Not at all useful”; 4 = “Very useful”). Following the combined intervention, participants will also rate treatment satisfaction: “How satisfied are you with the skills that this program has taught you to manage your PTSD?” (1 = “Not at all satisfied”; 9 = “Very satisfied”); “How confident would you be in recommending this treatment to a friend who experiences similar problems?” (1 = “Not at all confident”; 9 = “Very confident”); followed by two questions about difficulty with the program and requesting additional feedback, which will require free text responses.

### Additional measures

**Life Events Checklist for DSM-IV** (LEC-IV) [54]. The LEC-IV will be used to screen for exposure to traumatic life events. The LEC-IV assesses exposure to 16 potentially traumatic events (PTEs) plus one “other” item to capture PTEs not included in other items. It requires respondents to code how each event was experienced (i.e. “It happened to me; I witnessed it; I learned about it; Not sure; Doesn’t apply to me”). It has sound test-retest reliability and convergent validity with comparable measures [55].

**Part 2 of the Life Events Checklist for DSM-5** (LEC-5) [56]. The LEC-5 will be used to obtain additional information about reported traumatic life events not obtained from LEC-IV, including brief description of event, time since trauma, traumatic event frequency.

**Beck Depression Inventory – Second Edition – Question 9** (BDI-II) [57]. The BDI-II is a 21-item self-report inventory that measures depressive symptoms. The BDI-II possesses high internal consistency, with alpha coefficients of 0.86 and 0.81 for psychiatric and non-psychiatric populations, respectively [57]. Only Question 9 from this questionnaire will be used to help determine suicidal ideation and suicide risk. Patients indicate their level of intent to commit suicide, on a 4-point scale (0 = “I don’t have any thoughts of killing myself”; 3 = “I would kill myself if I had the chance”).

**Dissociative Experiences Scale** (DES) [58]. This is a 28-item measure of dissociative symptoms. Respondents indicate the frequency of dissociative experiences on an 11-point scale, increasing in 10-point increments from 0 % (never) to 100 % (always). This measure has strong internal consistency and reliability over time [59].

**Skills of Cognitive Behaviour Therapy for PTSD Questionnaire**. This measure has been adapted from the Skills of Cognitive Therapy – Patient version [60]. It is a 20-item self-report measure designed to assess patients' understanding and use of basic CBT skills in the PTSD program. Ratings of patients' skill usage are made on a 5-point scale (1 = “Never”; to 5 = “Always or when needed”). Higher scores reflect greater patient subjective skill in applying CBT therapy principles for their PTSD.

**Likely Comorbid Diagnostic Status**. Likely comorbid generalised anxiety disorder (GAD), social phobia (SP), panic disorder, agoraphobia and obsessive compulsive disorder (OCD) status will be indexed by asking the initial enquiry question from the relevant module in the MINI.

**Standardised Assessment of Personality** – **Abbreviated Scale** (SAPAS) [61]. The SAPAS is an 8-item screen for DSM-IV personality disorder, with each item requiring a Yes/No response. It is a sensitive, specific measure of likely personality disorder [61].

### Interventions

#### ICBT – the PTSD program

The PTSD program consists of six online lessons in the form of a cartoon narrative that contains best practice CBT as well as regular homework assignments and access to supplementary resources. Lesson summaries and skills practice homework assignments are available for download by participants following each lesson. Homework tasks encourage practice of the skills covered in each lesson (see Table 2). The program is completed over 10 weeks. Participants will be encouraged to complete the program within this time and receive automated reminders to progress. There will be a 5-day minimum interval from completing one lesson before the next is accessible to encourage skills practice before continuing. However, participants can choose to log in at any time beyond this. Participants can also choose the duration they spend on each lesson and skills practice. Participants are encouraged to spend 3–4 hours per week on lesson content and associated skills practice. An outline of key skills and resources covered across the PTSD program is provided in Table 2. Lessons are followed by skills practice assignments completed independently by patients throughout the iCBT program. This model has strong empirical support for the effective treatment of depression, GAD, SP, PD with and without agoraphobia, and mixed anxiety and depression [62], and is already used by our online treatment service, THIS WAY UP Clinic at St Vincent’s Hospital Sydney, Australia. Courses for the treatment of these other disorders are currently available to patients and, to date, over 5000 patients have completed a course.

**Table 2:** Skills and additional resources covered over the posttraumatic stress disorder (PTSD) program by lesson

Lesson	Skills	Extra resources
1	• Psychoeducation on PTSD diagnosis and treatment	• Managing mood • Sleep • Information for family and friends
2	• Psychoeducation: o How avoidance maintains PTSD o Fight-or-flight response o Cognitive behavioural model of PTSD o About cognitive behavioural therapy (CBT) • Controlled breathing	• Progressive muscle relaxation • Structured problem solving
3	• Link between thoughts and feelings • Thought monitoring • Psychoeducation for written exposure and practice of writing a trauma narrative	• Labelling emotions
4	• Psychoeducation and practice on reading the trauma narrative • Thought challenging	• Identifying and challenging thoughts in key themes (e.g. safety, trust)
5	• Behavioural experiments • Situational exposure • Continued writing and review of the trauma narrative	• Assertive communication • Extra information for family and friends • Attention shifting
6	• Key skills review • Relapse prevention	None

### Participant monitoring

The Clinical Trials Manager of CRUfAD and a member of the research team will oversee participant monitoring. Symptom severity of the target disorder and general distress levels are measured before, during, and immediately upon completing the PTSD program based on empirically validated standardised internet-delivered symptom measures. Participants will be sent automated emails at the completion of each lesson to encourage skills practice. Patient queries throughout the program are primarily addressed by email contact from the clinician (AA) or the Clinical Trials Manager (JS). If clinically indicated, or if a patient’s K10 and/or PHQ9 or PCL-C scores deteriorate, the clinician makes telephone contact. If a participant misses a lesson, a member of the research team will send an email reminder (via the Virtual Clinic system) or will make telephone contact to remind the participant to complete the lesson.

The treatment group will be required to complete the iCBT program within 10 weeks to remain in the trial. Adherence is monitored throughout the program. Once enrolled, a participant can elect to discontinue at any time. A participant may be withdrawn from the trial (typically meaning course access will be closed) for the following reasons: lack of computer and internet access; change in prescribed medication for anxiety or depression; change in medication status of exclusion medications; suicidality or clinical risk; failure to complete baseline questionnaires; failure to commence the PTSD program. Lack of adherence throughout the iCBT program is not a specified reason for participant withdrawal, although a participant may withdraw voluntarily. Those in the waitlist control group will be required to complete questionnaires at weeks 1, 5 and 11 of the waitlist period to remain in the RCT. The waitlist control group will have access to the iCBT program immediately at the conclusion of the waitlist period. All reasons for withdrawal status will be documented.

To promote participant retention, participants are reminded that data collection is an important aspect of research and enables the research team to track their progress and to evaluate the program. Any adverse events will be reported to the Director of CRUfAD (GA) and to the Human Research Ethics Committee (HREC) of St Vincent’s Hospital, the responsible body for initiating a clinical trial audit.

### Data management

All data will be collected via Virtual Clinic software and stored on a secure Virtual Clinic server. All information collected by the software is coded with either a participant identification number or an email address to facilitate data-to-patient matching. Clinical information not obtained from the online application, including diagnostic status using the MINI, is collected by interview via telephone and stored in written format in a secure location at CRUfAD. Any identifiable information collected remains confidential, except as required by law. Only members of the site (CRUfAD) research team will have access to participant information and data in order to monitor patient progress. During data analysis, re-identifiable data (i.e. coded data) will be used. At study completion, non-identifiable data will be written to a password-protected database. All data will be extracted from the Virtual Clinic servers in the form of an Excel-compatible file to be transferred to the IBM Statistical Package for the Social Sciences (IBM SPSS, IBM Corp., Armonk, NY, USA) by a member of the research team.

Participants will be informed in writing that the research team plans to disseminate the trial results in peer-reviewed scientific publications and presentations. Participants are informed that in any such dissemination, their anonymity will be maintained.

Participants will be sent (via email) a written summary of the results in lay terms following completion of the trial study phase.

### Statistical methods

Power calculations were informed by calculation of effect size data from Spence et al. [20], providing a between-group effect corresponding to Hedges’ g of 0.47. The minimum sample size for each group (alpha set at 0.05, power at 0.80) was identified as 72, but at least 10 % more will be recruited to hedge against expected attrition. All analyses will be conducted at conclusion of the trial period (i.e. after all participants in the treatment group have completed the iCBT program). Significance testing of group differences regarding demographic data and pre-treatment measurements will be conducted using analysis of variance (ANOVA) and χ2 where the variables consist of nominal (or categorical) data. Intent-to-treat (ITT) mixed models using restricted maximum likelihood (REML) estimation will be used to account for missing data due to participant drop-out. Mixed models do not assume that the last measurement is stable (the last observation carried forward assumption) [63]. REML models are appropriate for RCTs with multiple time points and pre-to post-only designs [64]. The assumption that data are missing at random (MAR) will be evaluated using binary logistic regression to predict drop-out (0 = no drop-out, 1 = drop-out) and by comparing these two groups on baseline measures. Significant effects will be followed up with pairwise contrasts comparing mean pre-treatment to mean post-treatment scores. Complete-case analyses of the primary hypotheses using data from participants who complete all six lessons of the PTSD program will also be conducted. The effect of potential treatment moderators (e.g. time post-trauma, number of traumatic events) will be evaluated by including baseline variables of interest as a covariate and interaction term in separate mixed models analyses. Analyses will be performed in IBM SPSS version 21. Effect sizes will be calculated between groups (Hedges’ g) and within groups (Cohen’s d, adjusting for the repeated measures correlation) using the pooled standard deviation and adjusted for sample size.

Mediation analyses will be used to examine the association between change in PTSD symptoms over treatment and change in each of IU and emotion regulation in separate analyses. Tests of the indirect effects (mediation) will be conducted using PROCESS [65]. This method was chosen over the causal steps approach [66] based on recent research advocating the use of modern statistical approaches to quantify intervening variable models [67]. As recommended, particularly for small samples, estimates of indirect effects will be generated using bootstrapping analysis see [68, 69]. Bootstrapping is a nonparametric resampling method that generates an estimate of the indirect effect, and does not require assumptions about the shape of the sampling distribution that underlie the Sobel test. In bootstrapping analysis, the most stringent test of an indirect effect (mediation) is if the 95 % bias corrected and accelerated confidence intervals for the indirect effect do not include the value of 0. When zero is outside of the 95 % confidence interval estimate, the indirect effect is declared statistically different from zero at *p* < 0.05 (two-tailed), indicating that the effect of the independent variable on the dependent variable is contingent upon the effect of the proposed mediator [68]. In the current study, PROCESS for SPSS will be used to estimate 5000 bias-corrected bootstrap 95 % confidence intervals.

### Ethics and dissemination

Staff associated with the project are aware of, and will adhere to, the National Statement on Ethical Conduct in Human Research (National Health and Medical Research Council, 2007). The current trial protocol has been approved by the Human Research Ethics Committee of St Vincent’s Hospital, Sydney, Australia. The trial is registered as ACTRN12614001213639.

All members of the research team who provide intellectual input to the trial design, execution, or write-up will be acknowledged as an author on any publications.

## Discussion

The current RCT will test the efficacy of an iCBT intervention for PTSD. It seeks to replicate previous efficacy findings [20, 23] of iCBT for PTSD in a formally assessed PTSD sample from the general population. Moreover, it will examine the relationship between PTSD symptom change following treatment and changes in IU and emotion regulation. As such, results from the proposed RCT may point to future lines of enquiry into the role of these constructs in the mechanism of PTSD symptom change during CBT.

Notwithstanding the potential utility of the proposed RCT, the following potential limitations are noted. The control group in the current study is a waitlist control, rather than an active treatment control. Therefore, it will not be possible to eliminate a potential placebo effect. Moreover, the results will not speak to the relative utility of iCBT for PTSD compared to an alternative treatment approach or modality. These will be questions for future research. Further, while the trial will examine potential change in IU and emotion regulation ability, the iCBT program was not written to specifically target either of these constructs. As such, results will not permit definitive conclusions as to how treatment components impact these constructs. The current RCT will use DSM-IV PTSD diagnostic criteria and associated standardised assessment instruments, as validated DSM-5 measures were not available when recruitment began for the current project. While items reflecting the three additional DSM-5 PTSD symptoms from the PCL-5 have been included, it is important to note that formal diagnostic and associated self-report measures do not measure DSM-5 PTSD criteria specifically. We note that while having different clinicians conduct pre-treatment and follow-up interviews controls for bias, it may introduce inter-rater variability.

Finally, history of child sexual and/or physical abuse is not an exclusion criterion in the current study. This approach was undertaken to maximise the generalisability of the study findings, given the prevalence of early life abuse histories in those suffering from PTSD e.g. [5]. It is noted that emerging evidence suggests emotion regulation skills training may be required prior to trauma-focused treatment for survivors of child trauma in order to optimise their potential benefit from trauma-focused treatment e.g. [31]. Accordingly, some national treatment guidelines suggest a period of emotion regulation or stabilisation prior to trauma-focused CBT for survivors of child abuse or other repeated/prolonged exposure to trauma [10, 11]. However, there is evidence that trauma-focused treatment may have utility for such people even in the absence of emotion regulation training [38, 70, 71]. Indeed, in a treatment-seeking sample with PTSD, Jerud et al. [32] found no baseline differences in emotion regulation between those with and without history of child abuse. They further observed equivalence in improved emotion regulation between these groups following treatment, despite no prior emotion regulation training. Notwithstanding, it is acknowledged that omission of emotion regulation training may limit the potential success of the current iCBT program for some survivors of child trauma.

## Trial status

This article was first submitted on 19 June 2015. To date, 26 participants have met eligibility requirements. The first round of applications opened on 12 September 2014 and the first participant was enrolled on 13 September. Data collection aims to be complete in September 2016.

## Abbreviations

ANOVA: Analysis of variance
BDI-II: Beck Depression Inventory – Second Edition
CBT: Cognitive behaviour therapy
CEQ: Credibility and Expectancy/Satisfaction Questionnaire
CRUfAD: Clinical Research Unit for Anxiety and Depression
DES: Dissociative Experiences Scale
DSM-IV: Diagnostic and Statistical Manual of Mental Disorders, Fourth Edition
DSM-5: Diagnostic and Statistical Manual of Mental Disorders, Fifth Edition
ERQ: Emotion Regulation Questionnaire
GAD: Generalised anxiety disorder
GAD-7: Generalised Anxiety Disorder 7-Item Scale
HREC: Human Research Ethics Committee
iCBT: Internet-delivered CBT
ITT: Intent-to-treat
IU: Intolerance of uncertainty
IUS-12: Intolerance of Uncertainty Scale 12-item version
K10: Kessler Psychological Distress Scale 10-item version
LEC-IV: Life Events Checklist for DSM-IV
LEC-5: Life Events Checklist for DSM-5
MAR: Missing at random
MINI: Mini International Psychiatric Interview
OCD: Obsessive compulsive disorder
PCL-C: PTSD Checklist – Civilian version
PCL-5: PTSD Checklist for DSM-5
PHQ-9: Patient Health Questionnaire 9-item version
PTE: Potentially traumatic event
PTSD: Posttraumatic stress disorder
RCT: Randomised controlled trial
REML: Restricted maximum likelihood estimation
SAPAS: Standardised Assessment of Personality – Abbreviated Scale
SP: Social phobia
SPIRIT: Standard Protocol Items: Recommendations for Interventional Trials guidelines
SPSS: Statistical Package for the Social Sciences
SUDOR: Service Utilisation and Days Out of Role Questionnaire
